# Supplementary figures and images for: An Automated Dashboard to Improve Laboratory COVID-19 Diagnostics Management
Source: Front Digit Health. 2021 Dec 6;3:773986. doi: 10.3389/fdgth.2021.773986 (PMC8685224; doi:10.3389/fdgth.2021.773986)

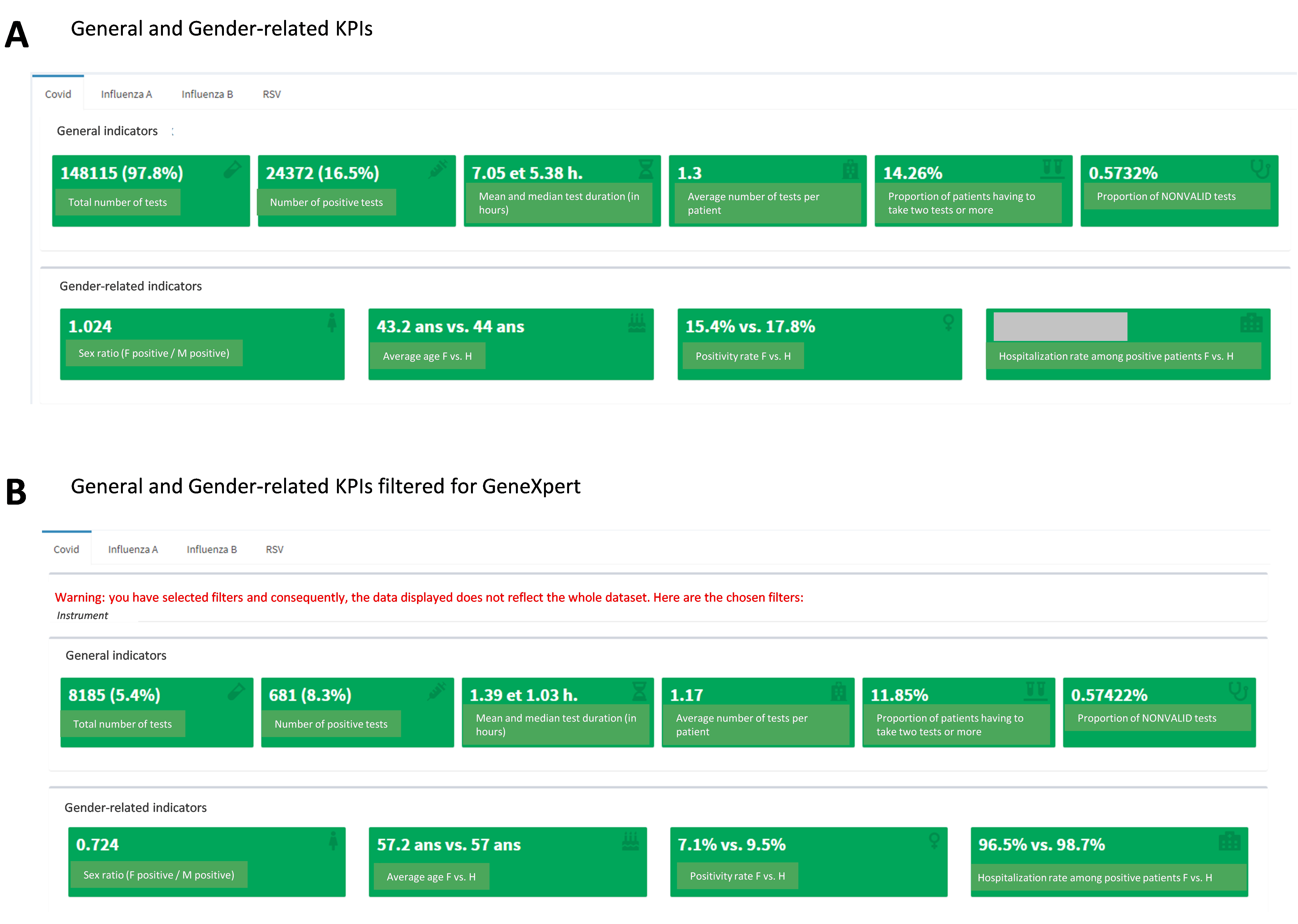

Supplement: Supplementary Figure 1 — Screen shots of the Filter page. (A) Number of tests and test results per date of reception. (B) Positivity rate per date of reception. [file Image_1.TIF]

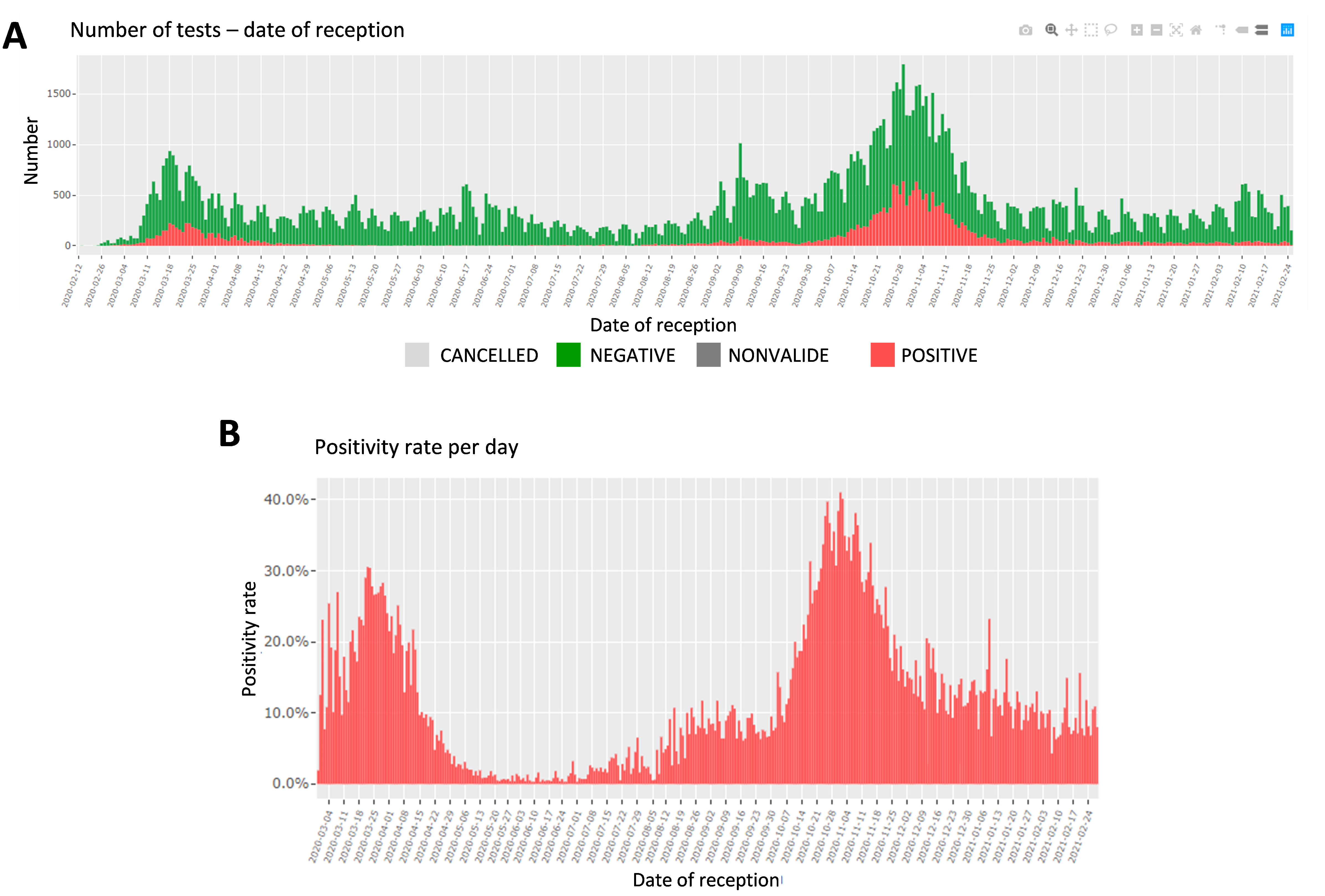

Supplement: Supplementary Figure 2 — Screen shots of the Filter page. (A) Number of tests and test results per date of reception. (B) Positivity rate per date of reception. [file Image_2.TIF]

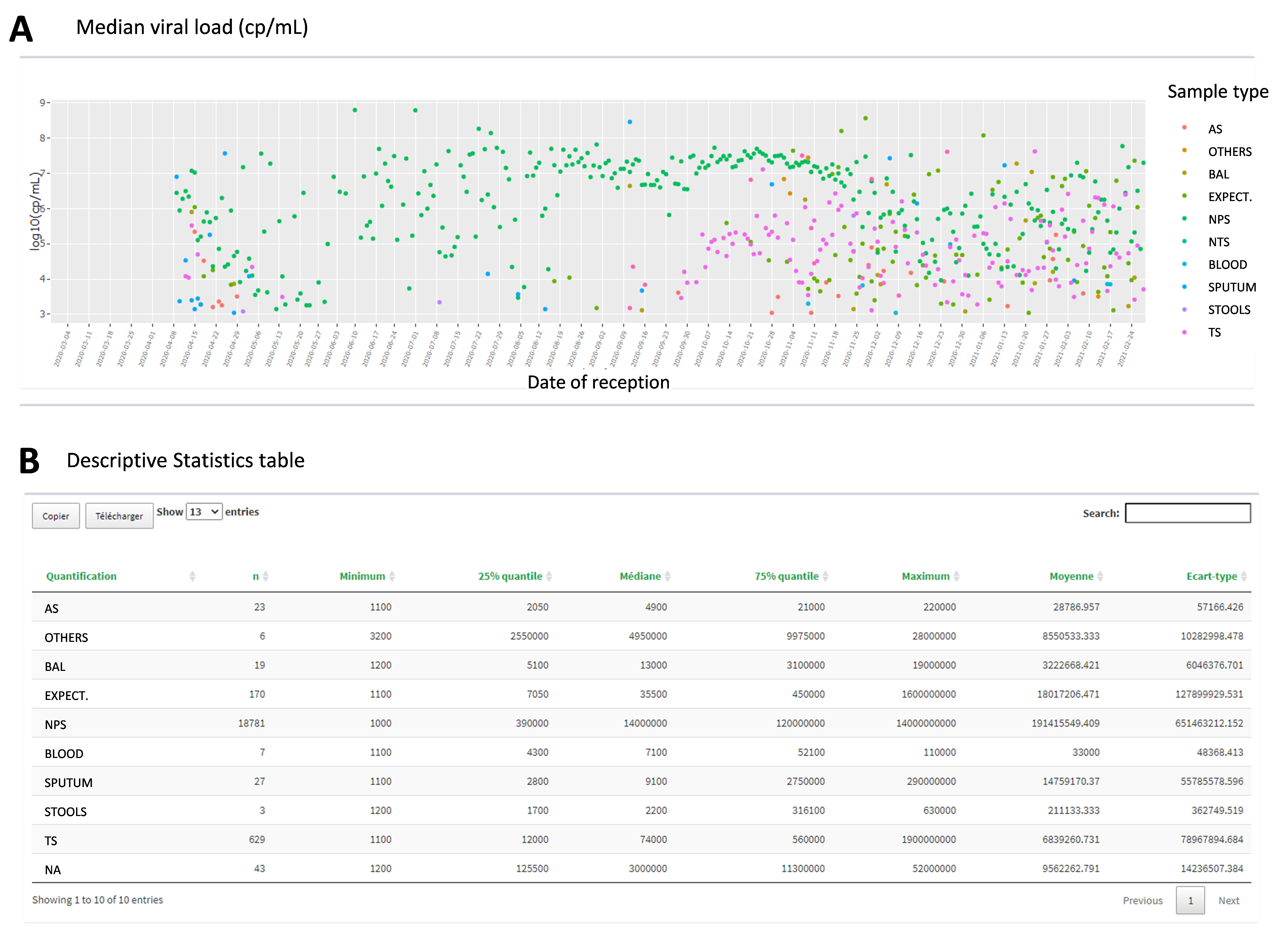

Supplement: Supplementary Figure 3 — Screen shots of the Filter page. (A) Viral load time series plot per sampling type, per date of reception and (B) descriptive statistics table. AS, anal swab; BAL, bronchoalveolar lavage; EXPECT, expectoration; NPS, nasopharyngeal swab; TS, throat swab; NA, non-applicable. [file Image_3.TIF]

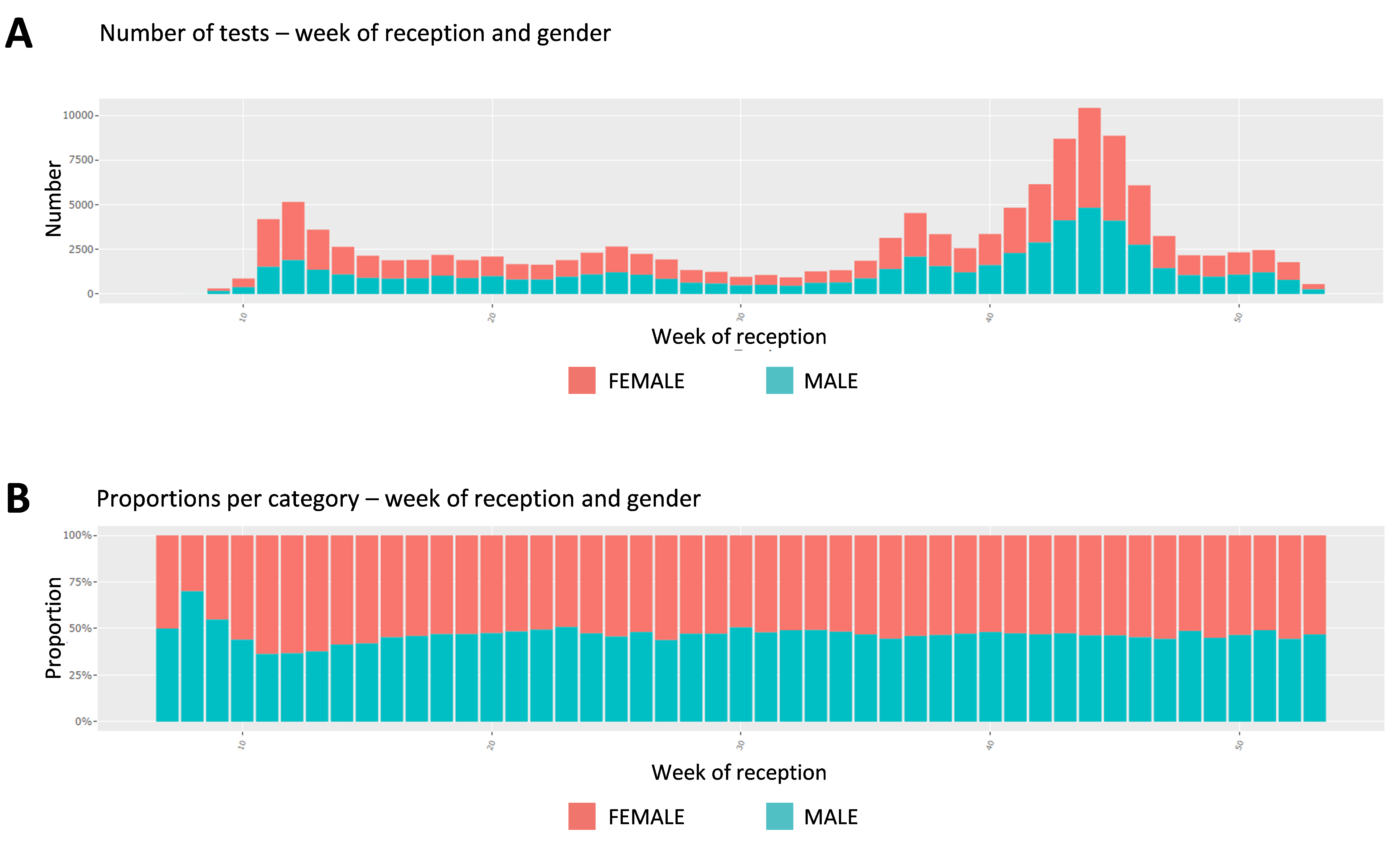

Supplement: Supplementary Figure 4 — Screen shots of the Comparison page that allows 182 different combinations, focus on gender KPI. (A) Number of tests per gender, per week of reception and (B) proportion of each gender, per week of reception. [file Image_4.TIF]
